# Choroid plexus volumes and auditory verbal learning scores are associated with conversion from mild cognitive impairment to Alzheimer's disease

**DOI:** 10.1002/brb3.3611

**Published:** 2024-07-02

**Authors:** Michael J. Pearson, Ruth Wagstaff, Rebecca J. Williams, for the Alzheimer's Disease Neuroimaging Initiative

**Affiliations:** ^1^ Faculty of Health Charles Darwin University Darwin Northern Territory Australia

**Keywords:** Alzheimer's disease, choroid plexus, magnetic resonance imaging, mild cognitive impairment, MRI, progressive MCI, RAVLT, Rey's Auditory Verbal Learning Test

## Abstract

**Purpose:**

Mild cognitive impairment (MCI) can be the prodromal phase of Alzheimer's disease (AD) where appropriate intervention might prevent or delay conversion to AD. Given this, there has been increasing interest in using magnetic resonance imaging (MRI) and neuropsychological testing to predict conversion from MCI to AD. Recent evidence suggests that the choroid plexus (ChP), neural substrates implicated in brain clearance, undergo volumetric changes in MCI and AD. Whether the ChP is involved in memory changes observed in MCI and can be used to predict conversion from MCI to AD has not been explored.

**Method:**

The current study used data from the Alzheimer's Disease Neuroimaging Initiative (ADNI) database to investigate whether later progression from MCI to AD (progressive MCI [pMCI], *n* = 115) or stable MCI (sMCI, *n* = 338) was associated with memory scores using the Rey Auditory Verbal Learning Test (RAVLT) and ChP volumes as calculated from MRI. Classification analyses identifying pMCI or sMCI group membership were performed to compare the predictive ability of the RAVLT and ChP volumes.

**Finding:**

The results indicated a significant difference between pMCI and sMCI groups for right ChP volume, with the pMCI group showing significantly larger right ChP volume (*p* = .01, 95% confidence interval [−.116, −.015]). A significant linear relationship between the RAVLT scores and right ChP volume was found across all participants, but not for the two groups separately. Classification analyses showed that a combination of left ChP volume and auditory verbal learning scores resulted in the most accurate classification performance, with group membership accurately predicted for 72% of the testing data.

**Conclusion:**

These results suggest that volumetric ChP changes appear to occur before the onset of AD and might provide value in predicting conversion from MCI to AD.

## Graphical abstract

Choroid plexus (ChP) volumes and Rey Auditory Verbal Learning Test—Immediate (RAVLT‐I) scores were compared between participants who later progressed from MCI to Alzheimer's disease (pMCI) and those who remained stable (sMCI). The right ChP was larger in the pMCI group, and the left ChP and RAVLT‐I were the best‐performing classifiers for predicting group membership.

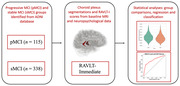

## 1 INTRODUCTION

Alzheimer's disease (AD) is a multifactorial neurodegenerative disorder that can progress from cognitively normal to mild cognitive impairment (MCI) to AD (Dubois et al., 2016).

As a syndrome defined by clinical and functional criteria, a diagnosis of MCI requires both subjective and objective cognitive changes. Memory can be affected, although other cognitive domains including executive functioning or language may be impaired (Petersen et al., 2014). While there are different underlying causes of MCI, AD pathology can be one etiology (DeCarli, 2003). Therefore, MCI can be the prodromal or transition stage between cognitively normal and AD, providing an important time period in which early detection and intervention can occur (Anderson, 2020). Reported conversion rates of MCI to AD differ depending on study, cohort, and population. Conversion rates of 24% and 34% from meta‐analyses performed in different countries have been reported (McGrattan et al., 2022; Mitchell & Shiri‐Feshki, 2009). Identification of those who are at high risk of conversion during the critical MCI period is essential for optimal disease intervention, management, and possible prevention (Aisen et al., 2011).

Neuroimaging biomarkers sensitive to pathophysiological changes indicating conversion from MCI to AD are of intense interest to the medical and scientific community (Feng et al., 2022; MacDonald & Pike, 2021; Rye et al., 2022; Veitch et al., 2019). Magnetic resonance imaging (MRI) offers excellent spatial resolution and tissue contrast, and the wide availability of large MRI datasets allows for the implementation of machine learning approaches for prediction (MacDonald & Pike, 2021). Classification algorithms trained on MRI data have an approximate 70% accuracy rate in identifying participants who will convert to AD from MCI (Battista et al., 2020; Salvatore et al., 2016). Such algorithms are often trained on features derived from gray matter, such as whole‐brain and regional gray matter volumes (Guo et al., 2014) and cortical thickness (MacDonald et al., 2019; Shin et al., 2021). Recently, there has been increased interest in imaging indicators that are sensitive to dysregulated brain clearance in AD (Tadayon, Pascual‐Leone, et al., 2020), which may provide essential new information to improve prediction accuracy.

While brain inflammation is a normal product of aging, AD is associated with significantly more pervasive inflammation (Balusu et al., 2016; Čarna et al., 2023). Inflammation weakens the immune system and slows the clearing of waste toxins from the brain (Li et al., 2023). The cerebrospinal fluid (CSF) provides waste removal and protection to the brain (Jessen et al., 2015). The CSF is primarily produced by the choroid plexus (ChP), which also acts as a barriers separating CSF from other fluids within the brain. The ChP is located bilaterally within the third, fourth, and lateral ventricles (Hablitz & Nedergaard, 2021; Lun et al., 2015). In AD, the ChP changes shape and increases in volume as the CSF decreases its protection of the brain from inflammation (Balusu et al., 2016; Liu et al., 2022). Recent work has demonstrated associations between increased ChP volumes and CSF proteins, potentially reflecting disrupted clearance of protein aggregates in AD (Tadayon, Pascual‐Leone, et al., 2020). Moreover, ChP enlargement has been reported in MCI patients compared to cognitively normal controls (Choi et al., 2022). A longitudinal investigation has shown that ChP volumes increase over time in older adults and are significantly larger in subjects who convert to MCI or AD, compared to subjects who remain cognitively normal (Novakova Martinkova et al., 2023). These findings suggest that ChP volumes increase in the prodromal stages of AD and therefore might represent important neuroimaging biomarkers that can be used to predict conversion to AD in patients living with MCI.

Another predictor of conversion from MCI to AD is memory impairment (Belleville et al., 2017). Indeed, memory loss is widely regarded as the initial symptom of AD (Talwar et al., 2021). Subsequently, declines are experienced in other cognitive domains such as language, visuospatial abilities, and executive functions (Jahn, 2013; Knopman et al., 2021). Subjective memory complaints and impaired performance detected by neuropsychological testing are key diagnostic features of MCI (Albert et al., 2011). The Rey Auditory Verbal Learning Test (RAVLT) is a neuropsychological tool that has been previously implemented in diagnosing AD (Estévez‐González et al., 2003). It is sensitive to various measures related to memory function, such as learning, delayed memory recall, false positives, and percentage forgetting (Schmidt, 1996). An episodic verbal list learning measure, RAVLT—Immediate (RAVLT‐I), has been established to show superior performance in differentiating participants with a diagnosis of stable MCI and those with progressive MCI who later convert from MCI to a diagnosis of AD (Moradi et al., 2017; Rye et al., 2022). Moradi and colleagues (2017) reported that the RAVLT‐I had significantly higher accuracy in predicting conversion to AD than other RAVLT measures, with a 72% success rate. These authors further demonstrated that RAVLT‐I scores can be predicted from whole‐brain gray matter density maps quantified from structural MRI. However, the ChP is not classified as gray matter and was therefore not included in this prior work. Further investigation is therefore required to establish whether there is a relationship between ChP volume and memory performance in the prodromal stage of AD.

The present study aimed to establish whether ChP volume is related to memory performance in MCI and can identify those who will later convert from MCI to AD. This was achieved by utilizing data from a longitudinal study, enabling the comparison of participants placed into two separate groups: those with stable MCI (sMCI) who remained stable in their diagnosis of MCI over the course of the study period and those with progressive MCI (pMCI) who converted from MCI to AD during the study. At the time of testing, all participants were diagnosed with MCI. Longitudinal follow‐up data enabled long‐term outcomes relating to AD conversion. The first research aim was to determine whether ChP volumes and RAVLT‐I scores differed between participants with sMCI and pMCI. The second research aim was to establish whether there was a relationship between ChP volumes and RAVLT‐I scores. The third research aim was to investigate the predictive ability of ChP volumes and RAVLT‐I scores in MCI by determining whether these can accurately predict later conversion to AD.

## 2 MATERIALS AND METHODS

Data used in the present work were obtained from the Alzheimer's Disease Neuroimaging Initiative (ADNI) database (adni.loni.usc.edu). The ADNI was launched in 2003 as a public–private partnership, led by Principal Investigator Michael W. Weiner, M.D. The primary goal of ADNI has been to test whether serial MRI, positron emission tomography (PET), other biological markers, and clinical and neuropsychological assessment can be combined to measure the progression of MCI and early AD. For up‐to‐date information, see www.adni‐info.org. Importantly, the longitudinal study design allows for predictions about AD conversion to be made, which was critical to the current research question.

### 2.1 Participants

Only participants aged between 55 and 91 years old who are conversationally fluent in English or Spanish were eligible to participate in an ADNI study (Weiner et al., 2015). The ADNI study and all study personnel comply with the Regulations for the Protection of Human Subjects of Research as well as all federal regulations of good clinical practice. Written informed consent was obtained from the participants for the storage and distribution for research purposes of all images and cognitive testing data.

The ADNI clinical raters assigned participants as either cognitively normal, MCI, or AD based on years of education, the Wechsler Memory Scale—Revised, the Mini‐Mental State Exam (MMSE), and a clinical dementia rating (Petersen et al., 2010). For the present study, only participants who were assigned MCI on their baseline session were considered. The criteria for diagnosing MCI by ADNI clinical raters are as follows: (1) participant must express a subjective memory concern or report concern to a study partner or clinician; (2) an educationally adjusted cutoff score from the Logical Memory II subscale Delayed Paragraph Recall, Paragraph A only, from the Wechsler Memory Scale—Revised (score <11 for those with ≥16 years of education; score ≤9 for those with between 8 and 15 years of education; score ≤6 for those with ≤7 years of education); (3) Clinical Dementia Rating of 0.5 and Memory Box score of at least 0.5; and (4) general cognition performance that is insufficient for a diagnosis of AD. The clinical protocols implemented by the ADNI studies can be found at https://adni.loni.usc.edu/methods/documents/.

For these MCI participants, all follow‐up sessions across all ADNI studies (ADNI1, ADNI2, ADNIGO, ADNI3) were carefully evaluated. Participants who recorded an MCI diagnosis for all of their ADNI sessions, with at least 24 months of follow‐up data from the baseline session, were allocated to the sMCI group. Allocation to the pMCI group was through examination of all participants whose diagnoses changed from MCI to AD in a subsequent session for any ADNI study. For example, a participant with a consistent MCI diagnosis over the course of ADNI1 and ADNI2, but who converted to AD during the ADNI3 study, would be allocated to the pMCI group. Participants were excluded if they (a) had a change of diagnosis back to MCI in any follow‐up sessions, (b) had MRI and neuropsychological testing more than 1 year apart, or (c) converted to AD in less than 1 year from the baseline testing session. This resulted in a total of 115 participants allocated to the pMCI group and 338 to the sMCI group. The ADNI roster identification numbers of all participants utilized in the present work can be found in Table S1. Participant demographic data can be found in Table 1.

**TABLE 1:** Demographic, magnetic resonance imaging (MRI), and neuropsychological testing information for the two mild cognitive impairment (MCI) groups.

	sMCI (*n* = 338)	pMCI (*n* = 115)
Demographic information		
Age (years)	72.70 (± 7.55)	73.76 (± 7.62)
Age range (years)	55–91	55–90
Number of females	138 (40.8%)	53 (46.09%)
Education (years)	16.07 (± 2.62)	16.08 (± 2.62)
Brain volumes		
Estimated total intracranial volume (mm^3^)	1,527,494 (± 167,879.00)	1,530,178.69 (± 173,089.22)
Left choroid plexuses (mm^3^)	1424.06 (± 608.64)	1433.10 (± 467.99)
Right choroid plexuses (mm^3^)	1188.56 (± 411.09)	1289.57 (± 412.89)
Neuropsychological testing		
RAVLT‐I	37.33 (± 10.81)	30.55 (± 8.73)
MRI information		
Field strength (1.5 T, 3 T)	95 (28%), 243 (72%)	28 (24%), 87 (76%)
Vendor (GE, Phillips, Siemens)	116 (34%), 44 (13%), 178 (53%)	33 (29%), 19 (16%), 63 (55%)

Abbreviations: pMCI, progressive MCI; RAVLT‐I, Rey Auditory Verbal Learning Test—Immediate; sMCI, stable MCI.

### 2.2 MRI analyses

For each participant, images were obtained from the earliest possible MRI scan session. For most participants, this was the baseline or initial scan. The date of the MRI acquisition was obtained from the DICOM header information and carefully checked to ensure that it aligned with the corresponding ADNI session with an MCI diagnosis and to determine the differences between the MRI acquisition date, the neuropsychological testing date, and conversion to AD date for the pMCI group.

All image analyses were performed on three‐dimensional T_1_‐weighted structural images, either magnetization‐prepared rapid gradient echo (MP‐RAGE) or inversion recovery spoiled gradient (IR‐SPGR). Images were collected on either General Electric (GE) Healthcare, Philips Medical Systems, or Siemens Medical Solutions MRI scanners. As data were obtained from all ADNI studies (ADNI, ADNI2, ADNIGO, ADNI3), there were variations in vendor, field strength, and imaging parameters. Summary information regarding the MRI scanners can be found in Table 1 for both groups. MR images were downloaded from the ADNI database as DICOMs and converted to NIFTI format for segmentation (Li et al., 2016).

**FIGURE 1:**
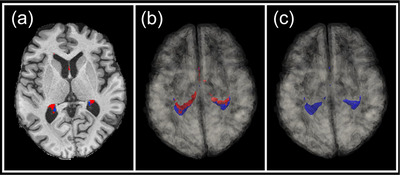
Example choroid plexus (ChP) segmentations from one participant (stable MCI group). The FreeSurfer ChP segmentations are overlaid in red, and Gaussian mixture model (GMM) segmentations are shown in blue in axial (a) and three‐dimensional rendered (b, c) views. The final segmentations used in all analyses in the present work were from the GMM only, shown in blue in panel (c).

Whole‐brain automatic segmentation was initially performed on each participant's T_1_‐weighted image using FreeSurfer Version 7.4.1 (https://surfer.nmr.mgh.harvard.edu). This included the *recon‐all* function, which generates subcortical brain masks that can be used to calculate the volume of the bilateral ChP (Fischl et al., 2002). However, recent work by Tadayon, Moret, et al. (2020) introduced a more accurate method to obtain ChP volumes of the lateral ventricles compared to FreeSurfer alone. This unsupervised machine learning approach is based on the Gaussian Mixture Model (GMM), which is applied to all voxels within the lateral ventricles. This is achieved by utilizing segmentations of the lateral ventricles generated by FreeSurfer. These authors assessed their GMM method and compared it to FreeSurfer, with manual segmentations of the ChP as the ground truth. The GMM produced significantly more accurate segmentations of the ChP than FreeSurfer alone (Tadayon, Moret, et al., 2020). The MRI data used in this validation included the ADNI dataset, incorporating images from cognitively normal, MCI, and AD participant groups. Furthermore, the accuracy of the GMM in segmenting the ChP has been independently verified (Jeong et al., 2023), highlighting the robustness of this algorithm. In the present study, after all images had been processed in FreeSurfer, the GMM was applied to obtain the final ChP volumes. Only ChP volumes from the left and right lateral ventricles are included in the GMM. The implemented GMM code was downloaded from the author's GitHub repository (https://github.com/EhsanTadayon/choroid‐plexus‐segmentation) and run using Python. Example segmentations of the ChP from the present work from both FreeSurfer and GMM are demonstrated in Figure 1.

### 2.3 Neuropsychological testing

All ADNI participants undergo in‐depth neuropsychological assessment, with a broad array of cognitive functioning assessed. The present study focused on the RAVLT, a list learning task with 15 largely unrelated concrete nouns with limited semantic associations. The list is read in order with deviations for the first five trials, after which a second list of 15 largely unrelated words is introduced. The test takes approximately 10–15 min to complete with the administrators following a script that is provided to them to ensure that all tests are reliable (Schmidt, 1996). The RAVLT‐I measure is scored by summing the number of words recalled in trials 1–5. For all participants, the RAVLT‐I score was obtained from the neuropsychology testing session closest in date to the MRI acquisition.

### 2.4 Statistical analyses

To address the first and second research aims, group comparisons and regression analyses were performed. All statistical analyses were performed using SPSS version 29. Variables including age, sex, and years of education have previously been shown to be a significant predictor of conversion to AD and were therefore accounted for in the statistical analyses (Fritsch et al., 2002; Visser & Verhey, 2008). This was achieved by comparing age and years of education between the two groups using independent samples *t*‐tests and by including these variables in the regression analyses.

Left and right hemispheric ChP volumes were normalized by the estimated intracranial volume, which was generated from the FreeSurfer analysis. All ChP volumes included in statistical analyses were normalized as the ratio of the estimated intracranial volume. The left and right ChPs were analyzed separately. The first analyses compared the pMCI and sMCI groups for ChP volumes and RAVLT scores using independent samples *t*‐tests. Paired samples *t*‐tests were performed to determine if there was a difference between left and right ChP volumes for each group.

The relationships between RAVLT‐I scores and left and right ChP volumes were assessed in multiple regression analyses with the RAVLT‐I as the outcome variable and ChP volume, age, sex, and years of education as the predictor variables. Separate regressions were performed with the left and right ChPs as predictor variables. One participant (from the sMCI group) was removed from all RAVLT analyses as an outlier with an RAVLT‐I score less than 3 standard deviations from the mean.

### 2.5 Classification

To address the third research aim, classification was performed using open‐source Python packages including jupyter, matplotlib, numpy, pandas, seaborn, and scikit‐learn (Pedregosa et al., 2011). Two supervised machine learning binary classification approaches were implemented—one using the *k*‐nearest neighbors (*k*NN) algorithm and another using Random Forest. These were performed to compare the accuracy of group membership (sMCI or pMCI) classification from ChP volumes and RAVLT‐I scores. To avoid misclassification due to sample size differences, the number of individuals in the sMCI group was reduced to match the pMCI group using random selection. Each *k*NN classifier was trained on 70% of the data, with 10‐fold cross‐validation performed to determine *k*. For Random Forest, hyperparameter tuning was performed to find the optimal model. The predictor variables included the left and right ChP volumes and RAVLT‐I data individually, as well as all combinations of features resulting in seven different classifiers: left ChP volume, right ChP volume, and RAVLT‐I individually; left + right ChP volume; RAVLT‐I + left ChP volume; RAVLT‐I + right ChP volume; and RAVLT‐I + left ChP volume + right ChP volume. Classification metrics including accuracy, specificity, sensitivity, and area under the receiver operating characteristic (ROC) curve enabled comparisons between models in terms of accuracy.

## 3 RESULTS

### 3.1 Participants

Independent samples *t*‐tests showed that there were no differences between the sMCI and pMCI groups in terms of age (*p* = .13) or number of years of education (*p* = .80). Table 1 shows the means and standard deviations of demographic data for the two groups.

For the pMCI group, the number of days to conversion to AD was calculated by taking the difference between the MRI acquisition date used in the present analyses and the first ADNI session date with an AD diagnosis. The mean difference was 1264.4 (± 765.5) days, with a range of 386–3730 days. The mean difference between the MRI acquisition date and neuropsychological testing date for the pMCI group was 40.2 (± 51.5) days, with a range of 0–359 days. For the sMCI group, the mean difference between MRI acquisition and neuropsychological testing was 39.7 (± 46.1) days, with a range of 0–348 days.

### 3.2 Group comparisons

**FIGURE 2:**
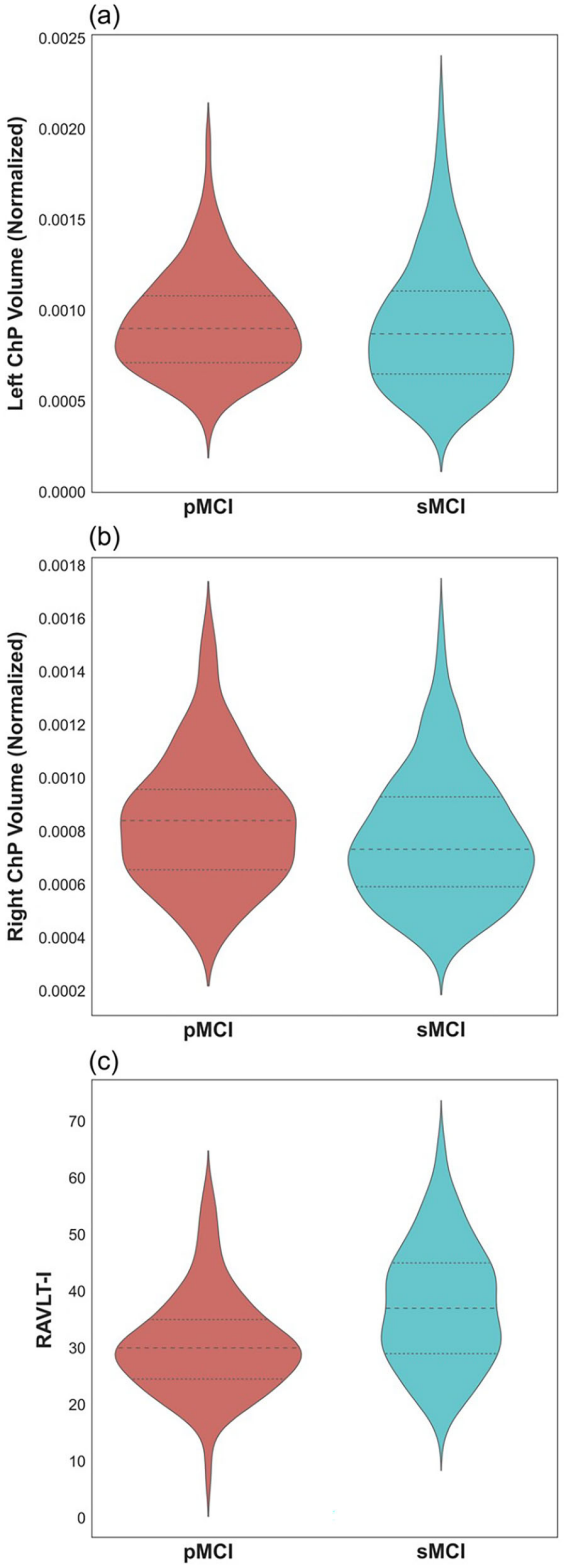
Violin plots with quartiles showing the distribution of (a) left and (b) right choroid plexus (ChP) volumes normalized by estimated intracranial volume, and (c) Rey Auditory Verbal Learning Test—Immediate (RAVLT‐I) scores for the progressive MCI (pMCI) (left, red) and stable MCI (sMCI) (right, blue) groups. There was a significant difference between the two groups for RAVLT‐I scores (*p* < .001) and right ChP volumes (*p* = .01).

The independent samples *t*‐tests comparing the two groups showed a significant difference for right ChP, with right ChP volumes larger for the pMCI group (*t*(451) = −2.54, *p* = .01, 95% confidence interval [CI] [−0.116, −0.015]). There was no difference between the two groups for the left ChP (*p* = .83). The paired samples *t*‐tests indicated that the left ChP was larger than the right ChP for both sMCI and pMCI groups (both *p *< .001). Figure 2 shows violin plots with the mean, quartiles, and range of values of left and right ChP volumes.

For the independent samples *t*‐test performed on RAVLT‐I scores, Levene's Test for Equality of Variances was significant, indicating heterogeneity of variances. Therefore, the results of the Welch *t*‐test are reported. There was a significant difference between the two groups for RAVLT‐I scores, with the sMCI group having higher RAVLT‐I scores than the pMCI group (*t*(441.7) = 6.76, *p* < .001, 95% CI [4.93, 8.87]). The mean RAVLT‐I scores are shown in Table 1, and the means, quartiles, and distributions of scores are displayed in Figure 2.

### 3.3 Regression

The assumptions for multiple regression were assessed and met for all reported regression analyses, including the presence of outliers and multicollinearity. When the left ChP volume was included as a predictor variable with age, sex, and years of education, and the RAVLT‐I was used as the outcome variable, the overall model was significant when all participants (collapsed over group) were included (*F*(4, 447) = 16.20, *p* < .001, *R* ^2^ = .13, adjusted *R* ^2^ = .12). However, the left ChP was not a significant predictor variable in this analysis (*t* = −0.99, *p* = .33). Age, sex, and years of education all significantly predicted the RAVLT‐I score (*p* < .001, *p* < .001, and *p* = .02, respectively).

**FIGURE 3:**
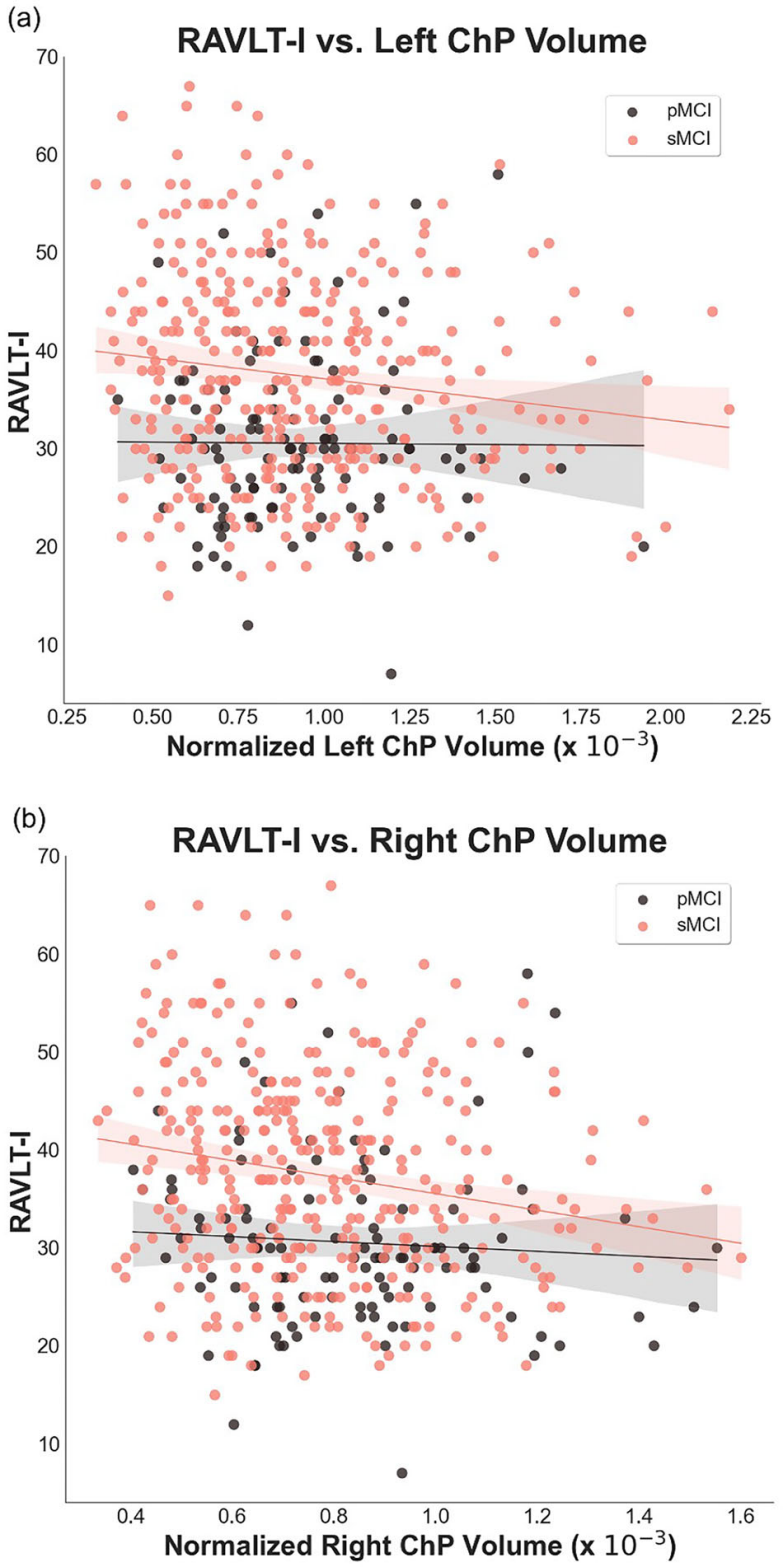
Scatterplots showing regression lines for Rey Auditory Verbal Learning Test—Immediate (RAVLT‐I) score and left choroid plexus volume (a) and right choroid plexus volume (b) for both groups (progressive MCI [pMCI] in black and stable MCI [sMCI] in pink).

A separate regression analysis was performed for the sMCI group only with the left ChP as a predictor variable (along with age, sex, and years of education); the overall model remained significant (*F*(4, 332) = 19.15, *p* < .001, *R* ^2^ = .19, adjusted *R* ^2 ^= .18). However, the left ChP remained a nonsignificant predictor (*p* = .32). When only the pMCI group was analyzed, the regression with the left ChP as a predictor variable was nonsignificant. The scatterplot in Figure 3a shows the relationship between the RAVLT‐I and left ChP for both groups.

For the analyses with the right ChP volume, age, sex, and years of education as predictor variables and RAVLT‐I as the outcome variable, the overall model was significant when all participants (collapsed over group) were included (*F*(4, 447) = 17.16, *p* < .001, *R* ^2^ = .13, adjusted *R* ^2^ = .12). The right ChP was a significant predictor of the RAVLT‐I score (*t* = −2.1, *p* = .04, 95% CI [−8348.1, −244.7]). Age, sex, and years of education were also significant predictors (*p* < .001, *p *< .001, and *p* = .02, respectively).

When the regression analysis was performed only on the sMCI group with the right ChP, age, sex, and years of education as predictor variables, the overall model remained significant (*F*(4, 332) = 19.59, *p* < .001, *R* ^2^ = .19, adjusted *R* ^2^ = .18). However, the right ChP was no longer a significant predictor (*t *= −1.56, *p* = .12). The regression with the right ChP as a predictor variable was nonsignificant when only the pMCI group was analyzed. The scatterplot in Figure 3b reveals the relationship between the RAVLT‐I and right ChP for both groups.

### 3.4 Classification

**FIGURE 4:**
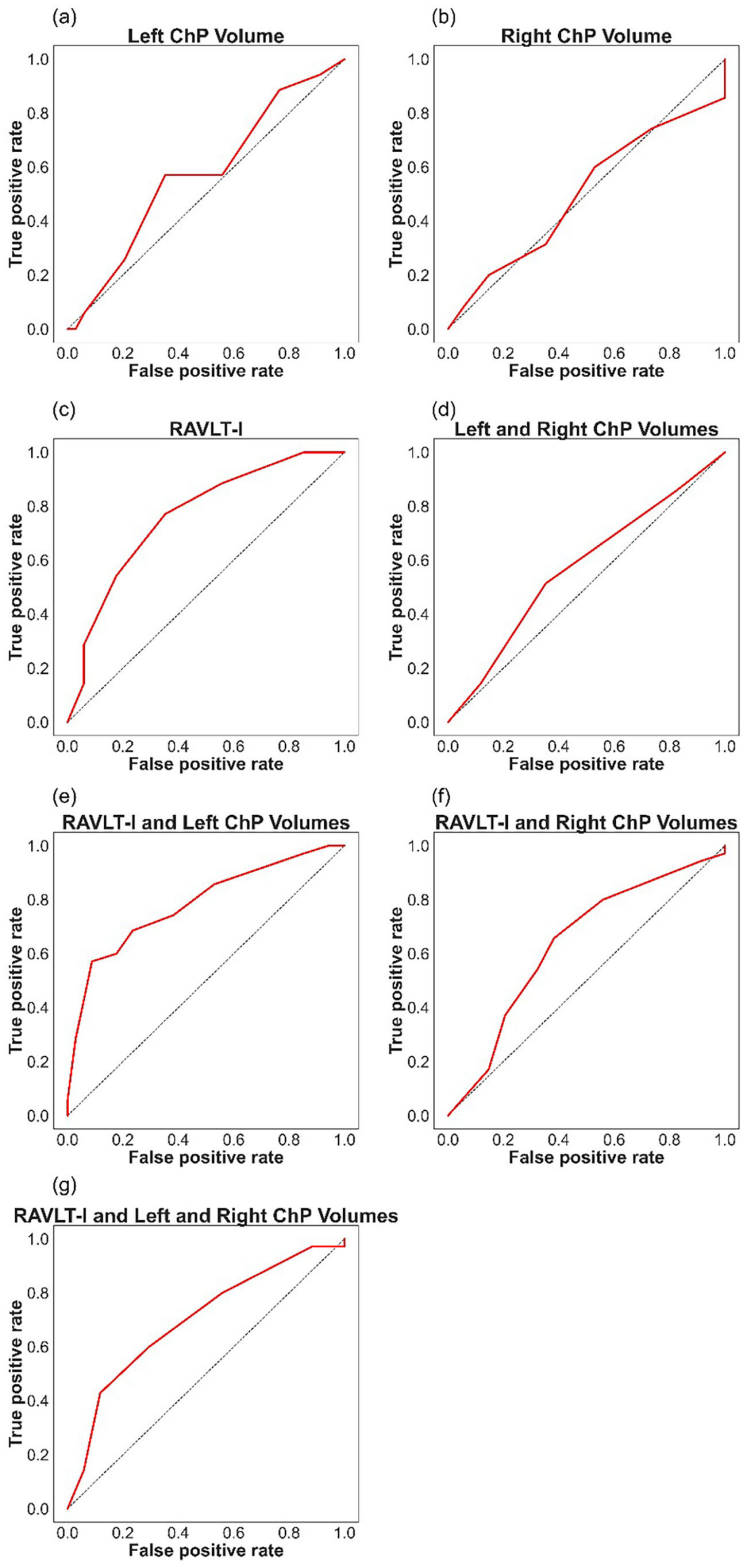
Receiver operating characteristic (ROC) curves for *k*NN classifiers used to predict group membership (progressive MCI or stable MCI) from (a) left choroid plexus (ChP) volumes, (b) right ChP volumes, (c) Rey Auditory Verbal Learning Test—Immediate (RAVLT‐I) scores, (d) left and right ChP volumes, (e) RAVLT‐I scores and left ChP volumes, (f) RAVLT‐I scores and right ChP volumes, and (g) RAVLT‐I scores and left and right ChP volumes. The best‐performing classifier was (e), with a 72% accuracy rate.

The 10‐fold cross‐validations performed determined the *k* for each of the seven *k*NN classifiers; all *k* were either 3, 7, or 11. These are shown in Table 2 with the performance metrics of the *k*NN and Random Forest classifiers. Based on the *k*NN metrics, the predictors that resulted in the best‐performing classifier for predicting group membership were the combination of RAVLT‐I + Left ChP volume, with an accuracy rate of 72% (shown in Figure 4e). When looking at individual predictors only, the RAVLT‐I outperformed both the left and right ChP volumes, with both hemispheres performing very poorly. However, the addition of left ChP volume to the RAVLT‐I score very slightly improved the accuracy, specificity, and area under the ROC curve. This can be observed from the ROC curves for each of the seven classifiers displayed in Figure 4. The sensitivity was superior with RAVLT‐I as a lone predictor variable. The results were similar when Random Forest classification was implemented. With this classifier, the predictors that resulted in the best‐performing classifier for predicting group membership were the combination of RAVLT‐I + Left ChP volume, with an accuracy rate of 70%. For the single predictors, RAVLT‐I scores outperformed left and right ChP volumes in terms of accuracy.

**TABLE 2:** Performance metrics of the *k*NN and Random Forest (RF) classifiers used to predict group membership (stable mild cognitive impairment or progressive mild cognitive impairment).

	Predictor variables	Accuracy	Sensitivity	Specificity
*k*NN				
	Left ChP volume (*k* = 7)	0.61	0.57	0.65
	Right ChP volume (*k* = 7)	0.54	0.60	0.47
	RAVLT‐I (*k* = 7)	0.71	0.77	0.65
	Left + Right ChP volumes (*k* = 3)	0.58	0.51	0.65
	RAVLT‐I + Left ChP volume (*k* = 11)	0.72	0.69	0.76
	RAVLT‐I + Right ChP volume (*k* = 7)	0.64	0.66	0.62
	RAVLT‐I + Left + Right ChP volumes (*k* = 7)	0.65	0.60	0.71
RF				
	Left ChP volume	0.59	0.68	0.59
	Right ChP volume	0.56	0.62	0.50
	RAVLT‐I	0.65	0.53	0.77
	Left + Right ChP volumes	0.55	0.59	0.54
	RAVLT‐I + Left ChP volume	0.70	0.58	0.78
	RAVLT‐I + Right ChP volume	0.67	0.51	0.82
	RAVLT‐I + Left + Right ChP volumes	0.64	0.61	0.67

Abbreviations: ChP, choroid plexus; RAVLT‐I, Rey Auditory Verbal Learning Test—Immediate.

## 4 DISCUSSION

This study aimed to explore the relationships between memory performance and ChP volumes in MCI and determine whether these can be used to predict conversion to AD. To this end, the first research aim was to establish whether significant differences existed between participants with MCI who would later progress to AD (pMCI) and participants who remained stable in their MCI diagnosis (sMCI) in terms of memory performance measured with the RAVLT‐I and ChP volumes. Significant differences were found, with the pMCI group having significantly larger ChP volumes in the right hemisphere and significantly lower RAVLT‐I scores than the sMCI group. The second research aim sought to establish whether there was a relationship between RAVLT‐I scores and ChP volumes; this was largely unsupported by the present results, with the only significant relationship found between RAVLT‐I scores and right ChP volumes when all participants collapsed over the two groups were analyzed. The third research question asked whether group membership (sMCI or pMCI) could be predicted from ChP volumes and RAVLT‐I scores. Individually, the accuracy of group membership classification from ChP volume was poor. When combined with the RAVLT‐I, the left ChP showed reasonable classification performance and slightly improved classification performance of the RAVLT‐I alone. Overall, these results suggest that ChP volume in prodromal AD may be indicative of later conversion to AD, although the negative findings from the regression and classification analyses should also be taken into consideration.

Understanding the neurobiological underpinnings of memory changes in MCI is crucial for predicting disease progression and implementing treatment (Albert et al., 2011). Prior work has characterized volumetric changes in key memory regions in patients living with MCI. Medial temporal lobe regions including the entorhinal cortex and hippocampus have shown significant volume reductions in patients living with MCI compared to healthy controls (Pennanen et al., 2004). Atrophy of the medial temporal lobe is associated with reduced cognitive scores in MCI (van de Pol et al., 2007). More recently, it has been shown that the ChP undergoes morphological alterations in people living with MCI and AD (Choi et al., 2022), leading to the research question of whether ChP changes are associated with memory changes observed in these patients. While there is no evidence suggesting that the ChP is directly implicated in memory function, reduced capacity of the ChP may indirectly lead to memory impairment due to alterations in the brain environment. The main function of the ChP is to produce the CSF, which is critical for healthy brain function as it provides mechanical support, facilitates neuronal activity by transporting neuromodulators, and removes metabolic byproducts (Bjorefeldt et al., 2018; Kaur et al., 2016; Kelley, 2021). The present work did not find a significant linear relationship between ChP volume and RAVLT‐I score in sMCI and pMCI groups separately. While the overall regression models were significant for the sMCI group, the coefficient for the ChP predictor was not significant. For the sMCI group, this indicates that age and sex are better predictors of RAVLT‐I scores than ChP volumes. For the pMCI group, the overall regression models were nonsignificant, indicating that even age and sex were not significant predictors of the RAVLT‐I score. This discrepant finding between the two groups (sMCI and pMCI) might be due to the larger sample size of the sMCI group. These findings indicate that the question of whether ChP volume is associated with memory performance in these groups remains unclear. Another consideration of these nonsignificant findings is the choice of neuropsychological test. The RAVLT‐I was chosen as it has been used previously in research predicting conversion to AD in patients living with MCI (Mofrad et al., 2021; Moradi et al., 2017; Rye et al., 2022), and therefore it is the best choice of test to enable comparison. Prior work has reported negative correlations between ChP volume and cognitive test scores in AD (Choi et al., 2022), which was supported by the results of the present study but only when all MCI participants were analyzed together. Further work is warranted to determine whether there are other neuropsychological tests associated with ChP volume in sMCI and pMCI groups separately.

The present work reported significant differences between the pMCI and sMCI groups in terms of the RAVLT‐I score and right ChP volume. The group differences in RAVLT‐I scores were expected given prior findings (Moradi et al., 2017). However, the group difference in right ChP volume is a novel contribution to the literature. There was no difference between the pMCI and sMCI groups for the left hemisphere ChP volume; however, for both groups, the left hemisphere ChP was significantly larger than the right. Chronic immune responses are known to occur in AD (Kinney et al., 2018), and there is clear evidence implicating the ChP in this inflammation (Gião et al., 2022; Strominger et al., 2018). Inflammation appears to be associated with observed ChP volume changes in MCI and AD (Choi et al., 2022); however, it is unclear why a hemispheric difference would exist. When looking at Table 1, both groups showed larger left ChP than right ChP, but the pMCI group seemed to demonstrate relative enlargement of the right ChP. A speculative interpretation is that the left ChP is the first hemisphere to respond to inflammatory processes occurring in MCI, and the engagement of both hemispheres for the pMCI group might be indicative of stronger inflammation and risk of AD conversion. Asymmetry of the ChP has been found in the developing brain, with the left hemisphere larger than the right (Corballis, 2013). Whether this early life asymmetry is associated with later aging‐related inflammation is unclear. A counterargument to the biological perspective is that there is an MRI artifact affecting the volumetric analyses. However, support for this is weak as scanning took place on different MRI scanners across multiple sites. Importantly, the present findings are not the first to report laterality effects of the ChP. Consistent with the results presented here, one prior MRI study that collected independent data (i.e., was not affiliated with ADNI) reported a significant increase in right‐hemisphere ChP volume when comparing participants with complex regional pain syndrome to healthy controls (Zhou et al., 2015). Clearly, further work is required to understand why such laterality effects of the ChP are reported, and whether this is associated with immune surveillance and response.

A limitation of this study is the known heterogeneity of the MCI groups. Numerous studies have identified early and late subtypes of MCI based on neuropsychological testing scores (Aisen et al., 2010; Edmonds et al., 2019; Lin et al., 2022; Wei et al., 2018). In the present work, early and late MCI was not overtly differentiated, instead focusing on conversion to AD in keeping with comparable research focused on progressive and stable MCI (Mofrad et al., 2021; Moradi et al., 2017; Rye et al., 2022). The pMCI and sMCI groups may contain variance attributed to these MCI subtypes, which could be further differentiated to improve classification accuracy, should sample size permit. Another limitation of the present study that must be considered when interpreting the results is the known limitations associated with automatic segmentation. Diligence in ChP segmentation accuracy was undertaken by including an additional algorithm, the GMM. However, the initial estimates for this algorithm are based on FreeSurfer outputs. Furthermore, the normalization of the ChP was performed using FreeSurfer total intracranial volume segmentation estimates. Research has shown that FreeSurfer segmentations can be biased, particularly for subcortical structures (Srinivasan et al., 2020). A final study limitation is the chosen data and the possible information that could improve classification performance. There is a wealth of information that could be included to improve classification accuracy and prediction of cognitive function, such as more gray matter features from structural MRI (e.g., cortical thickness, whole‐brain gray matter parcellations), functional MRI (e.g., cerebral blood flow, functional connectivity, cerebrovascular reactivity), and CSF biomarkers quantifying AD pathology (e.g., beta‐amyloid concentration) (Fjell et al., 2010; MacDonald et al., 2020; Vemuri et al., 2018; Williams et al., 2023). In the present work, the poor classification performance of the ChP volumes alone, but the slight improvement in performance when left ChP volume was combined with the RAVLT‐I, indicates that exploring other features that could be used for improving classification accuracy in conjunction with ChP volume is a worthy future research endeavor.

In summary, the present work provides novel interpretations of the ChP as a potential biomarker for conversion from MCI to AD. ChP volume from the right hemispheric lateral ventricle showed significantly larger volumes in patients who later progressed from MCI to AD, compared to those who remained stable in their MCI diagnosis. However, the best group classification performance was found when the *k*NN classifier was trained on left ChP volumes and RAVLT‐I scores, despite very poor performance when the ChP volumes were the only features. While further research is required to determine the significance of these laterality differences, the presented results support the further exploration of the ChP, in conjunction with other pertinent imaging features and neuropsychological test scores, for understanding the processes underlying conversion from MCI to AD.

## AUTHOR CONTRIBUTIONS

**Michael J. Pearson**: Conceptualization; data curation; formal analysis; methodology; writing—review and editing. **Ruth Wagstaff**: Project administration; supervision; writing—review and editing. **Rebecca J. Williams**: Conceptualization; formal analysis; methodology; project administration; supervision; visualization; writing—original draft; writing—review and editing.

### PEER REVIEW

The peer review history for this article is available at https://publons.com/publon/10.1002/brb3.3611.

## Supporting information

Table S1. ADNI roster identification (RID) numbers and corresponding group (pMCI or sMCI) of all participants.

## Data Availability

All image and neuropsychological testing data were downloaded from the ADNI database (https://adni.loni.usc.edu/). The roster identification codes are in Table S1. The python code used for the analysis is publicly available via github: https://github.com/rjaydewilliams/ADNI_CHP_MCI.
