# Supplementary material for: Choroid plexus volumes and auditory verbal learning scores are associated with conversion from mild cognitive impairment to Alzheimer's disease
Source: Brain Behav. 2024 Jul 2;14(7):e3611. doi: 10.1002/brb3.3611 (PMC11219301; doi:10.1002/brb3.3611)
Supplement: Supplementary file 1 — Table S1. ADNI roster identification (RID) numbers and corresponding group (pMCI or sMCI) of all participants. [file BRB3-14-e3611-s001.docx]

| **RID**  Table S1. ADNI roster identification (RID) numbers and corresponding group (pMCI or sMCI) of all participants. | **GROUP** |
| --- | --- |
| 135 | pMCI |
| 160 | pMCI |
| 276 | pMCI |
| 285 | pMCI |
| 292 | pMCI |
| 361 | pMCI |
| 376 | pMCI |
| 448 | pMCI |
| 501 | pMCI |
| 552 | pMCI |
| 588 | pMCI |
| 605 | pMCI |
| 626 | pMCI |
| 671 | pMCI |
| 698 | pMCI |
| 702 | pMCI |
| 830 | pMCI |
| 896 | pMCI |
| 914 | pMCI |
| 925 | pMCI |
| 945 | pMCI |
| 994 | pMCI |
| 1030 | pMCI |
| 1032 | pMCI |
| 1074 | pMCI |
| 1078 | pMCI |
| 1080 | pMCI |
| 1097 | pMCI |
| 1106 | pMCI |
| 1117 | pMCI |
| 1186 | pMCI |
| 1318 | pMCI |
| 1351 | pMCI |
| 2087 | pMCI |
| 2106 | pMCI |
| 2155 | pMCI |
| 2220 | pMCI |
| 2248 | pMCI |
| 2316 | pMCI |
| 2373 | pMCI |
| 2380 | pMCI |
| 2381 | pMCI |
| 2398 | pMCI |
| 2403 | pMCI |
| 4015 | pMCI |
| 4030 | pMCI |
| 4035 | pMCI |
| 4042 | pMCI |
| 4058 | pMCI |
| 4079 | pMCI |
| 4102 | pMCI |
| 4114 | pMCI |
| 4131 | pMCI |
| 4157 | pMCI |
| 4167 | pMCI |
| 4171 | pMCI |
| 4188 | pMCI |
| 4203 | pMCI |
| 4243 | pMCI |
| 4250 | pMCI |
| 4263 | pMCI |
| 4271 | pMCI |
| 4272 | pMCI |
| 4293 | pMCI |
| 4303 | pMCI |
| 4324 | pMCI |
| 4331 | pMCI |
| 4346 | pMCI |
| 4363 | pMCI |
| 4365 | pMCI |
| 4406 | pMCI |
| 4415 | pMCI |
| 4426 | pMCI |
| 4462 | pMCI |
| 4521 | pMCI |
| 4538 | pMCI |
| 4582 | pMCI |
| 4584 | pMCI |
| 4596 | pMCI |
| 4597 | pMCI |
| 4623 | pMCI |
| 4631 | pMCI |
| 4668 | pMCI |
| 4715 | pMCI |
| 4720 | pMCI |
| 4746 | pMCI |
| 4777 | pMCI |
| 4782 | pMCI |
| 4815 | pMCI |
| 4816 | pMCI |
| 4823 | pMCI |
| 4862 | pMCI |
| 4893 | pMCI |
| 4902 | pMCI |
| 4929 | pMCI |
| 5026 | pMCI |
| 5031 | pMCI |
| 5237 | pMCI |
| 6034 | pMCI |
| 6068 | pMCI |
| 6073 | pMCI |
| 6241 | pMCI |
| 6252 | pMCI |
| 6341 | pMCI |
| 6467 | pMCI |
| 6529 | pMCI |
| 6632 | pMCI |
| 6640 | pMCI |
| 6668 | pMCI |
| 6770 | pMCI |
| 6788 | pMCI |
| 6800 | pMCI |
| 6805 | pMCI |
| 6852 | pMCI |
| 6911 | pMCI |
| 135 | sMCI |
| 160 | sMCI |
| 276 | sMCI |
| 285 | sMCI |
| 292 | sMCI |
| 361 | sMCI |
| 376 | sMCI |
| 448 | sMCI |
| 501 | sMCI |
| 552 | sMCI |
| 588 | sMCI |
| 605 | sMCI |
| 626 | sMCI |
| 671 | sMCI |
| 698 | sMCI |
| 702 | sMCI |
| 830 | sMCI |
| 896 | sMCI |
| 914 | sMCI |
| 925 | sMCI |
| 945 | sMCI |
| 994 | sMCI |
| 1030 | sMCI |
| 1032 | sMCI |
| 1074 | sMCI |
| 1078 | sMCI |
| 1080 | sMCI |
| 1097 | sMCI |
| 1106 | sMCI |
| 1117 | sMCI |
| 1186 | sMCI |
| 1318 | sMCI |
| 1351 | sMCI |
| 2087 | sMCI |
| 2106 | sMCI |
| 2155 | sMCI |
| 2220 | sMCI |
| 2248 | sMCI |
| 2316 | sMCI |
| 2373 | sMCI |
| 2380 | sMCI |
| 2381 | sMCI |
| 2398 | sMCI |
| 2403 | sMCI |
| 4015 | sMCI |
| 4030 | sMCI |
| 4035 | sMCI |
| 4042 | sMCI |
| 4058 | sMCI |
| 4079 | sMCI |
| 4102 | sMCI |
| 4114 | sMCI |
| 4131 | sMCI |
| 4157 | sMCI |
| 4167 | sMCI |
| 4171 | sMCI |
| 4188 | sMCI |
| 4203 | sMCI |
| 4243 | sMCI |
| 4250 | sMCI |
| 4263 | sMCI |
| 4271 | sMCI |
| 4272 | sMCI |
| 4293 | sMCI |
| 4303 | sMCI |
| 4324 | sMCI |
| 4331 | sMCI |
| 4346 | sMCI |
| 4363 | sMCI |
| 4365 | sMCI |
| 4406 | sMCI |
| 4415 | sMCI |
| 4426 | sMCI |
| 4462 | sMCI |
| 4521 | sMCI |
| 4538 | sMCI |
| 4582 | sMCI |
| 4584 | sMCI |
| 4596 | sMCI |
| 4597 | sMCI |
| 4623 | sMCI |
| 4631 | sMCI |
| 4668 | sMCI |
| 4715 | sMCI |
| 4720 | sMCI |
| 4746 | sMCI |
| 4777 | sMCI |
| 4782 | sMCI |
| 4815 | sMCI |
| 4816 | sMCI |
| 4823 | sMCI |
| 4862 | sMCI |
| 4893 | sMCI |
| 4902 | sMCI |
| 4929 | sMCI |
| 5026 | sMCI |
| 5031 | sMCI |
| 5237 | sMCI |
| 6034 | sMCI |
| 6068 | sMCI |
| 6073 | sMCI |
| 6241 | sMCI |
| 6252 | sMCI |
| 6341 | sMCI |
| 6467 | sMCI |
| 6529 | sMCI |
| 6632 | sMCI |
| 6640 | sMCI |
| 6668 | sMCI |
| 6770 | sMCI |
| 6788 | sMCI |
| 6800 | sMCI |
| 6805 | sMCI |
| 6852 | sMCI |
| 6911 | sMCI |
